# Computational and *in vitro* Pharmacodynamics Characterization of 1A-116 Rac1 Inhibitor: Relevance of Trp56 in Its Biological Activity

**DOI:** 10.3389/fcell.2020.00240

**Published:** 2020-04-15

**Authors:** Nazareno González, Georgina A. Cardama, Patricio Chinestrad, Javier Robles-Valero, Sonia Rodríguez-Fdez, L. Francisco Lorenzo-Martín, Xosé R. Bustelo, Pablo Lorenzano Menna, Daniel E. Gomez

**Affiliations:** ^1^Laboratory of Molecular Oncology, National University of Quilmes, Bernal, Argentina; ^2^National Scientific and Technical Research Council (CONICET), Buenos Aires, Argentina; ^3^Molecular Pharmacology Laboratory, National University of Quilmes, Bernal, Argentina; ^4^Centro de Investigación del Cáncer, CSIC-University of Salamanca, Salamanca, Spain; ^5^Instituto de Biología Molecular y Celular del Cáncer, CSIC-University of Salamanca, Salamanca, Spain; ^6^Centro de Investigación Biomédica en Red de Cáncer (CIBERONC), CSIC-University of Salamanca, Salamanca, Spain

**Keywords:** small-molecule, docking, inhibitor, GTPases, cancer

## Abstract

In the last years, the development of new drugs in oncology has evolved notably. In particular, drug development has shifted from empirical screening of active cytotoxic compounds to molecularly targeted drugs blocking specific biologic pathways that drive cancer progression and metastasis. Using a rational design approach, our group has developed 1A-116 as a promising Rac1 inhibitor, with antitumoral and antimetastatic effects in several types of cancer. Rac1 is over activated in a wide range of tumor types and and it is one of the most studied proteins of the Rho GTPase family. Its role in actin cytoskeleton reorganization has effects on endocytosis, vesicular trafficking, cell cycle progression and cellular migration. In this context, the regulatory activity of Rac1 affects several key processes in the course of the cancer including invasion and metastasis. The purpose of this preclinical study was to focus on the mode of action of 1A-116, conducting an interdisciplinary approach with *in silico* bioinformatics tools and *in vitro* assays. Here, we demonstrate that the tryptophan 56 residue is necessary for the inhibitory effects of 1A-116 since this compound interferes with protein-protein interactions (PPI) of Rac1GTPase involving several GEF activators. 1A-116 is also able to inhibit the oncogenic Rac1^P29S^ mutant protein, one of the oncogenic drivers found in sun-exposed melanoma. It also inhibits numerous Rac1-regulated cellular processes such as membrane ruffling and lamellipodia formation. These results deepen our knowledge of 1A-116 inhibition of Rac1 and its biological impact on cancer progression. They also represent a good example of how *in silico* analyses represent a valuable approach for drug development.

## Introduction

Rho GTPases are molecular switches that cycle between two conformational states: an inactive GDP-bound form and an active GTP-bound form. This cycle is highly regulated by guanine nucleotide exchange factors (GEFs), which catalyze nucleotide exchange and mediate Rho GTPase activation, and GTPase-activating proteins (GAPs), which stimulate GTP hydrolysis to return the GTPases to the inactive, GDP-bound state. The active, GTP-bound Rho proteins bind preferentially to downstream effector proteins to engage the downstream biological responses (Jaffe and Hall, 2005; Bustelo et al., 2007; Bustelo, 2018). Additionally, Rho GTPases are regulated by a wide range of post-translational modifications, such as prenylation, proteolytic cleavage, methylation, phosphorylation, sumoylation, and ubiquitination, to ensure specific spatiotemporal activation (Bustelo et al., 2007; Rathinam et al., 2011).

Rho GTPases are readily activated by different stimuli that activate a wide variety of cell-surface receptors, including receptor tyrosine kinases (RTKs), G-protein-coupled receptors (GPCRs), cytokine receptors, integrins and cadherins (Bustelo, 2018). These stimulated receptors ultimately promote the exchange of GDP for GTP on Rho proteins, mainly by GEF activation. To date, more than 70 GEFs have been reported. Some of the most well described GEFs include Tiam1, Dbl, Vav family, P-Rex1, Dock-180 (Vigil et al., 2010).

Rac1 is one of the most studied members of Rho-GTPases family and controls fundamental cellular processes. Rac1 is a significant regulator in actin cytoskeleton reorganization, affecting endocytosis trafficking, cell cycle progression, cell adhesion, and migration (Etienne-Manneville and Hall, 2002; Bustelo et al., 2007). Accumulating evidence indicates that Rac1 is overexpressed and hyperactivated in a wide range of tumors and its influence on cytoskeleton remodeling affects key processes such as invasion, migration, and metastasis of cancer cells (Sahai and Marshall, 2002; Bustelo, 2018). Other GTPases, such as RhoA, RhoG, and Ccd42, have also been associated with cancer progression (Bustelo, 2018).

We have previously reported the rational design and development of the novel Rac1 inhibitor 1A-116. This compound was identified using a structure-based drug discovery approach (SBDD) that involves virtual library screening and docking using tryptophan 56 (W56) as a target of Rac1 3D structure. This amino acid is a crucial residue for Rac1 activation by different GEFs (Gao et al., 2001). Importantly, W56 residue is not unique to Rac1 protein. In fact, it is highly conserved in other members of the Rho GTPase family of proteins, such as Rac2/3 and RhoA/B/C/D/G (Haeusler et al., 2003). 1A-116 Rac1 inhibitor showed antitumoral activity *in vitro* on a wide variety of cancer types such as breast cancer (Cardama et al., 2014a; Gonzalez et al., 2017), glioblastoma (Cardama et al., 2014b) and acute myeloid leukemia (Cabrera et al., 2017). In this regard, we have already reported that 1A-116 has a profound effect on proliferation, migration, invasion, metastasis, apoptosis, and cell cycle arrest.

Protein flexibility is a fundamental requirement for most biological functions. Indeed, the use of a single protein structure in SBDD implies accepting the outdated lock-and-key model as the unique recognition process between protein and ligands. In contrast, considering the conformational diversity of a protein may improve the probability succeeding in discovering novel active compounds (Setiawan et al., 2018).

In this work, we show evidence of the mechanism of action involved in 1A-116 biological activity. Our results support the relevance reported of W56 residue for 1A-116 activity, confirming the previous SBDD approach used for its identification. We also carried out a detailed analysis of the conformational diversity of Rac1, considering all the available crystallographic structures in the Protein Data Bank (PDB). Using docking experiments, we analyzed the stability of Rac1-1A116 interactions. In addition, we evaluated the ability of 1A-116 to interfere with Rac1 protein-protein interactions (PPI) with a broad spectrum of GEFs involved in the tumoral phenotype. In particular, we showed that 1A-116 inhibits the interaction of Rac1 with Vav1, Vav2, Vav3, Tiam1, and Dbl. Finally, we showed for the first time that 1A-116 inhibits Rac1^P29S^, a rapid-cycling mutant of Rac1 that is frequently found in melanoma and other tumor types (Bustelo, 2018). We also demonstrate that 1A-116 prevented Rac1-regulated processes involved in the primary tumorigenesis and metastastic processes.

## Materials and Methods

### Cell Lines

COS-1 cells (ATCC^®^ CRL-1650^TM^) from African green monkey kidney fibroblast-like cell line were obtained from the American Type Culture Collection (ATCC). Cells were grown in Dulbecco’s modified Eagle’s medium (DMEM) (Life Technologies) supplemented with 10% heat-inactivated fetal bovine serum (FBS), 2 mM glutamine and 80 μg/ml gentamicin at 37°C in 5% CO_2_ atmosphere. Cell cultures were routinely subcultured twice a week by trypsinization and EDTA treatment (Gibco, Rockville, MD, United States), using standard procedures.

### Drugs

Chemo Argentina/Romikin S.A. kindly provided Rac1 inhibitor 1A-116 (Cardama et al., 2014a). The compound was synthesized under GMP conditions. Purity (HPLC): >99.3% 1A-116 was solubilized in aqueous solution at pH 5.5, by the addition of HCl 100 mM.

### Computational Conformational Analysis of Rac1 and Docking Experiments

The human Ras-related C3 botulinum toxin substrate 1 (Rac1) crystal structures were retrieved from the Protein Data Bank (PDB) (Berman et al., 2000). A total number of fifty-two (52) conformations, excluding structural mutants, were used for the analysis. The only single-point mutant conformations considered for the analysis were: the constitutively active mutant Q61L; the self-activating mutants P29S and F28L; and the dominant negative mutant T17N. A list of all the conformations used, together with a brief summary of their features, can be found in Supplementary Table 1. Root-mean-square deviation (RMSD) between all conformers and the *Z*-Scores derived from each C-alpha RMSD were calculated using a database of protein conformational diversity (CoDNaS) developed by Monzon et al., 2016. The 3D structure predictions of Rac1 W56F and CDC42 F56W single-point mutants were carried out by the I-TASSER server (Yang et al., 2015). The crystal structure of wild type Rac1 and CDC42 (1MH1 and 2QRZ) were obtained from the PDB and used as templates.

For the docking experiments, the pockets containing the residue W56 of Rac1 and F56 of Cdc42 were used as targets. The docking was centered on the C-alpha of this residue with a grid size of 14 1Å. AutoDock Vina was used as docking software (Trott and Olson, 2010). Each docking experiment was repeated one hundred times to determine the mean docking energy ± SD.

### Cell Proliferation Assays

COS-1 cells were plated in 96-wells plates and 24 h later were treated for 24 h with different concentrations of 1A-116. Cell growth was measured by colorimetric MTT assay (Sigma). The concentration producing 50% inhibition (IC50) was determined by non-linear regression function PRISM 6, Version 6.01 (GraphPad Prism6^®^ Software Inc., La Jolla, CA, United States). Results shown correspond to the average of three independent experiments.

### Serum-Response Assays

4 × 10^5^ COS-1 cells per well were plated into 6-well plates. Plasmids were diluted in OptiMEM (ThermoFisher Scientific), the transfection reagent Lipofectamine LTX (GIBCO/BRL, Gaithersburg, Maryland) was added to the DNA solution and incubated for 25 min at room temperature. Next, the DNA-Lipofectamine complexes were added to the cells and incubated overnight. Subsequently, the cells (in triplicate) were treated with vehicle (H_2_O) or 1A-116 50 μM for 24 h. Cells were then harvested and cell lysates were assayed for luciferase activity (Renilla normalized) utilizing the Dual-Luciferase Reporter Assay System (Promega) following the manufacturer’s instructions. The plasmids used for the GEFs experiments were: pCEFL-AU5, empty vector; pNM108, plasmid encoding the constitutively active version of Vav1 (Vav1 Δ1-189); pNM115, plasmid encoding the constitutively active version of Vav2 (Vav2 Δ1-186); pNM099, plasmid encoding the constitutively active version of Vav3 (Vav3 DH-PH-ZF); pcDNA.3-HA-C1199, plasmid encoding the constitutively active version of Tiam1 [Tiam1 C1199 (aa 391-1590)]; DBL-onco, plasmid encoding the constitutively active version of DBL (oncogenic Dbl, through truncation of the N-terminal 497 residues). For the GTPases experiments, the plasmids used were: pCEFL-AU5, empty vector; pCEFL-AU5-Rac1 Q61L, plasmid encoding the constitutively active version of Rac1; pJRC27, plasmid encoding the constitutively active version of Cdc42. Lastly, the plasmids used for specificity of 1A-116 were pNG01 and pNG02, encoding the Rac1 W56F Q61L and Cdc42 F56W Q61L mutants, and the Rac1 P29S mutants generated by site-directed mutagenesis procedures described below.

### Site-Directed Mutagenesis

Site-directed mutagenesis was carried out using the QuikChange kit according to the manufacturer’s instructions. To generate the vector encoding AU5-Rac1 W56F Q61L (pNG01), we used the pCEFL-AU5-Rac1 W56F (pJRC27) plasmid as template and the oligonucleotides 5′-GAT ACA GCT GGA CTA GAA GAT TAT GAC-3′ (forward) and 5′-GTC ATA ATC TTC TAG TCC AGC TGT ATC-3′ (reverse). To generate the vector encoding AU5-Cdc42 F56W Q61L (pNG02), we used the plasmid pCEFL-AU5-Cdc42 Q61L (pJRC27) as template and the oligonucleotides 5′-CTC TTG GAC TTT GGG ATA CTG CAG G-3′ (forward) and 5′-CCT GCA GTA TCC CAA AGT CCA AGA G-3′ (reverse). To generate the Rac1 P29S mutants, we used the pCEFL-AU5-RAC1 WT plasmid as template (for the Rac1 wt P29S) and the pCEFL-AU5-RAC1 Q61L (for the Rac1 P29S Q61L) and the oligonucleotides 5′-AGT TAC ACA ACC AAT GCA TTT TCT GGA GAA TAT ATC CCT ACT GTC-3′ (forward) and 5′-GAC AGT AGG GAT ATA TTC TCC AGA AAA TGA TTG GTT GTG TAA CT-3′ (reverse). Oligonucleotides were purchased from Thermo Fisher Scientific. All plasmids were sequence-verified at the Genomics and Proteomics Facility of Centro de Investigación del Cáncer, Salamanca, Spain.

### Rac1 Pull Down Assay

COS-1 cells were plated in p100 dishes. Next day, cells were transfected with pCEFL-AU5 or pCEFL-AU5-Rac1 Q61L, using CaCl_2_ and 24 h later monolayers were treated or not with 1A-116 50 μM for another 24 h period. Monolayers were washed with PBS and lysed in 150-GPLB Buffer supplemented with a protease inhibitor cocktail. Lysates were clarified and the protein concentrations were normalized. An aliquot was removed for determination of total Rac1 and the rest was incubated with Glutathione Sepharose 4B Beads, coupled with bacterially expressed GST-PAK1. Bound complexes were washed with lysis buffer, resuspended in protein sample buffer, boiled and loaded onto a 12% SDS-PAGE gel. Proteins were transferred and blotted with mouse monoclonal antibody against Rac1 (Sigma). SDS-PAGE and Coomassie Blue staining was used to check the integrity of the purified GST-PAK1 fusion proteins.

### 3D Organotypic Cultures

2 × 10^5^ human keratinocytes Ker-CT cells were seeded onto polycarbonate inserts (ThermoFisher, Catalog No. 140620) and cultured for 2 days in CnT–Prime medium. When confluency was reached, medium was changed to 3D–Barrier (CellnTec, Catalog No. CnT–PR–3D) and the air–lift was performed according to the manufacturer’s instructions. 3D cultures were maintained for 11 days with three medium changes per week. Treatment with 1A-116 (100 nM) was performed on the sixth day post air-lift. 1A-116 concentration was selected based on the minor effect induced in the organotypic structures formed by control cells. Immunohistochemistry studies were performed in the Pathology Service of the Cancer Research Center (Salamanca, Spain), fixing the 3D cultures for 16 h at 4°C, filling the well and the interior of the insert with paraformaldehyde at 3.7%. The skin/membrane structure was cut and proceeded to inclusion in paraffin and subsequent staining with hematoxylin and eosin, following standard procedures.

### Immunofluorescent Staining for Confocal Imaging

COS-1 cells were grown on 6-well plates and transfected with the constitutively active Rac1 Q61L plasmid, tagged at its N-terminus with EGFP (pNM42) or the empty vector pEGFP-C1 (as control), using Lipofectamine 2000 (GIBCO/BRL, Gaithersburg, Maryland). To that end, we mixed 1 μg of plasmid and 3 μl of Lipofectamine in 100 μl of serum-free OptiMEM. The transfection mix was then added into each well, cells cultured for 24 h and trypsinized. We then seeded them onto polylysine-coated coverslips and treated cells with vehicle or 1A-116 50 μM for an additional 24 h. Upon culturing under indicated experimental conditions, cells were fixed with 3.7% paraformaldehyde and subjected to conventional immunofluorescence techniques. In brief, cells were permeabilized with Triton X-100 0.5% m/v in TBS [25 mM Tris-HCl (pH 8.0), 150 mM NaCl] during 10 min of gentle agitation. Permeabilization buffer was removed and cells were washed with TBS (3 times, for 5 min) and then coverslips were blocked for 10 min with blocking solution (2% bovine serum albumin (BSA), 0.1% m/v sodium azide, 0.1% m/v Tritón X-100, 25 mM Tris (pH 7.5, adjusted by the addition of HCl 1M) (2% m/v BSA TBS), on gentle agitation. To visualize the F-actin cytoskeleton, cells were subsequently incubated with Alexa Fluor 635-labeled phalloidin diluted 1:200 in blocking solution for 20 min, washed three times with TBS, and stained with DAPI to visualize nuclei. The stained preparations were mounted on microscope slides using Mowiol (Calbiochem). Samples were analyzed by confocal microscopy using a Leica SP5 confocal microscope with a 63x-objective (Leica).

### Statistical Analysis

Statistical analyses were carried out using PRISM 6 software, Version 6.01 (GraphPad Prism6^®^ Software Inc., La Jolla, CA, United States). Results of this work were expressed as mean ± SEM; mean ± SD; mean ± confidence interval (CI). For multi-group analyses, one- or two-way ANOVA was applied, followed by Tukey’s multiple posterior comparisons test, or 95% CI comparison, as appropriate. The normal distribution of the data was determined using the D’Agostino-Pearson normality test. In addition, homoscedasticity was evaluated with the Bartlett test. For the data that did not follow a normal distribution or when the homoscedasticity was not met, the Kruskal–Wallis test was performed. In all cases, the differences were considered statistically significant at *p* < 0.05.

## Results

### Drug-Like Properties of 1A-116

**FIGURE 1:**
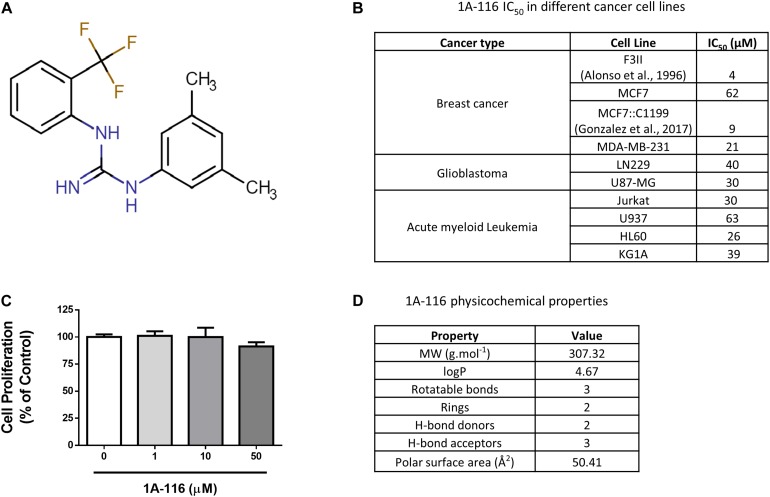
1A-116 is a Rac1 inhibitor. **(A)** Chemical structure of 1A-116. **(B)** Antiproliferative effects of 1A-116 1A-116 in different cancer cell lines. **(C)** Effects of 1A-116 in COS-1 cells used in SRE-activation assays. **(D)** Drug-like and physicochemical properties of 1A-116.

1A-116 (Figure 1A) is a small compound previously described by our group that was developed by a rational design approach using *in silico* virtual screening. In previous reports, we showed that 1A-116 was able to inhibit Rac1-GEF interactions reducing Rac1 activation levels and showing anti-proliferative effects on different cancer cell lines (Figure 1B; Cardama et al., 2014a, b; Cabrera et al., 2017; Gonzalez et al., 2017) but not in COS-1 cells used in the luciferase assays (Figure 1C). The drug-likeness of this small molecule compound meets Lipinski’s rules for small molecule drugs (Lipinski et al., 2001; Lipinski, 2004). As shown in Figure 1D, 1A-116 has a molecular weight of 307.32 g mol^–1^ with a predicted logP of 4.67. Moreover, it has two hydrogen bond donors and three hydrogen bond acceptors. In concordance with Veber’s rules (Veber et al., 2002), 1A-116 also meets the criteria of a small-molecule-drug-like compound with three rotatable bonds and a molecular polar surface area of 50.41 Å^2^, which is below the 140 Å^2^ recommended by Veber. These key physicochemical properties showed good drug-likeness and suggested good oral availability for this compound.

### *In silico* Conformational Diversity Analysis of Rac1 Crystallographic Structures

1A-116 family of small compounds was identified using an SBDD strategy, which involves the use of a single protein structure (PDB ID code 1MH1). Lately, it has been recognized that the conformational diversity of a protein is central to understanding protein function. This conformational diversity implies that proteins are not structured in a unique conformation and present differences between these conformations, having a potential impact on the success of the drug-target binding.

To further characterize the protein functionality of Rac1 and to take into account the conformational diversity of the Rac1 structure, all the crystal structures (fifty conformers without mutations) of Rac1 available in the PDB were retrieved.

**FIGURE 2:**
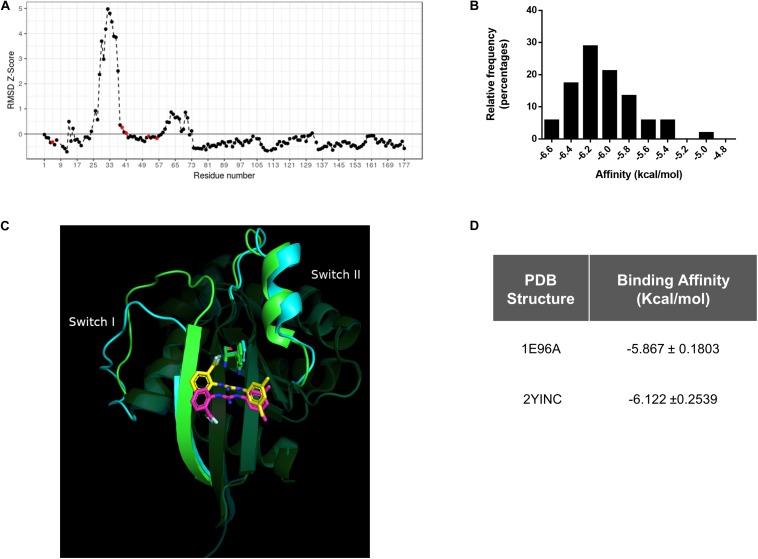
*In silico* Conformational diversity analysis of Rac1 crystallographic structures. **(A)** RMSD as *Z*-score for each pair of residues aligned in the maximum pair of conformers. Residues with a *Z*-Score lower than zero show a lower average mobility and therefore higher stability. Red dots represent the residues in close contact with 1A-116. **(B)** Distribution of binding affinities of 1A-116 against all the conformers of Rac1 by redocking experiments. In the X-axis the binding affinity in kcal/mol were plotted and in the Y-axis the relative frequency expressed in percentage of each binding affinity value (Mean -6.02 ± 0.315 kcal/mol) were plotted. **(C)** 1A-116 docked against the maximum RMSD pair of Rac1 conformers (1E96A gray, 2YINC white). The guanidine of 1A-116 establishes a H-Bond with Rac1 in both conformers. While the B ring is docked in the same position, the A ring is slightly rotated. **(D)** Binding affinity of 1A-116 against maximum pair of conformers obtained by redocking experiments.

By evaluating the RMSD scores, we could identify the pair of conformers of Rac1 that exhibited the maximal conformational diversity (1E96A vs. 2YINC, RMSD 2.5 Å). We also calculated the *Z*-Scores derived from Carbon-alpha RMSD per position of the maximum-pair of conformers, focusing on the binding site. We first surveyed the W56 residue and then in N52, S41, N39, and K5 residues since they are also involved in Rac1-1A116 interaction. The *Z*-Score distribution revealed reduced relative mobility of the residues involved in the interaction with 1A-116 (Figure 2A). These results indicate that W56 and the residues in close contact with 1A-116 are located in a relatively low mobility region within the Rac1 structure, contributing to the idea that W56 is an appropriate residue to target with small molecules to interfere Rac1-GEF interaction.

We further performed an exhaustive re-docking analysis to assess the interaction of 1A-116 with all the available X-ray structures of Rac1 deposited in the PDB. Autodock Vina was used to calculate the interaction of 1A-116 with all Rac1 conformers. The results showed that 1A-116 is predicted to bind with high affinity, with a mean affinity of −6.02 ± 0.315 kcal/mol (Figure 2B), suggesting that 1A-116 binds stably to all the reported conformational states of Rac1.

Furthermore, we calculated the RMSD scores between the best-docked positions of 1A-116 to the maximum pair of Rac1 structures to evaluate the binding mode of 1A-116. As shown in Figure 2C, 1A-116 interacts with both conformers through the same residues. The H-Bond with W56, which is crucial for the interaction, is present in both conformers. We also analyzed the position of 1A-116 with each Rac1 conformer and obtained an RMSD score of 4.24 Å, with a similar affinity in both cases (-5.867 ± 0.1803 kcal/mol to 1E96A, −6.122 ± 0.2539 kcal/mol to 2YINC) (Figure 2D). While the 2-trifluoromethylphenyl group (ring A) has an RMSD score of 5.51 Å, both the 3,5-dimethylphenyl group (ring B) and the guanidine are docked in a more similar position, with an RMSD score of 1.35 Å and 1.47 Å. This finding shows that the ring B and guanidine, which provides the H-Bond between 1A-116 and Rac1, are docked similarly in the maximum RMSD pair. In addition, the rotatable bonds near to ring A allows 1A-116 to adopt a stable position in both conformers.

### Tryptophan 56 of Rac1 Is Required for the Inhibitory Effects of 1A-116

**FIGURE 3:**
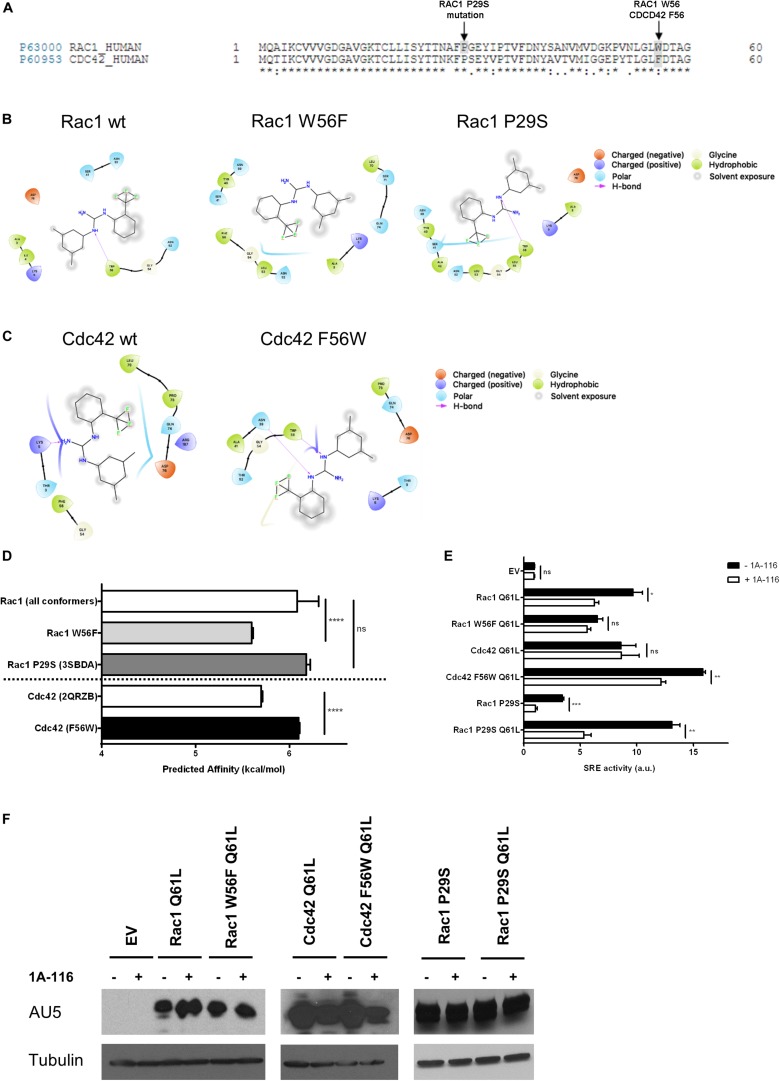
The W56 amino acid residue of Rac1 is necessary for the inhibitory effects of 1A-116. **(A)** Alignment of amino acid sequences of Rac1 (Uniprot ID p63000) and Cdc42 (Uniprot ID p60953). The shaded areas in gray mark the difference in the aminoacid 56, a key residue in Rac1-activation mediated by different GEFs and the Rac1 P29S mutation. **(B)** 2D representations of 1A-116 interaction with Rac1 WT and mutants (W56F and P29S). **(C)** 2D representations of 1A-116 interaction with Cdc42 WT and F56W mutant. **(D)** Predicted affinity (kcal/mol) of 1A-116 for different Rac1 and Cdc42 structures using AutoDock Vina software. Each docking experiment was repeated one hundred times to determine the mean docking energy ± SD. ANOVA; ns, not statistically significant; ****p < 0.0001. **(E)** COS-1 cells were co-transfected with active versions of Rac1 (Q61L), Cdc42 (Q61L), and the mutants versions of Rac1 (W56F Q61L, P29S, and P29S Q61L) and Cdc42 (F56W Q61L), together with the SRE-Luc and Renilla reporter plasmids. Cells were treated (+1A-116, white) or not (-1A-116, black) with 1A-116 50 μM and luciferase activity was measured. **(F)** Western blot expression detection of transfected exogenous proteins. The expressed protein is reported and the primary antibodies used (WB) were: α-AU5 for EV, Rac1 Q61L, Cdc42 Q61L, Rac1 W56F Q61L, Cdc42 F56W Q61L, Rac1 P29S, Rac1 P29S Q61L and α-Tubulin as a loading control. Error bars, SEM *t* test; ns, not statistically significant; *p < 0.05; **p < 0.01; ***p < 0.001; vehicle vs. 1A-116 for each protein. Luciferase expression was relativized to Renilla expression value (transfection control) and values reported are shown as an increase in activity with respect to the mean luciferase expression of the empty vector (EV), in arbitrary units (a.u.). Results are representative of at least three independent experiments.

Rac1 and Cdc42 share approximately 70% sequence homology, although they differ in specific key residues (Figure 3A). For example, the tryptophan residue of position 56 (W56) of Rac1 is substituted by a phenylalanine (F56) residue in the case of Cdc42 (shaded area in gray). As we mentioned above, the W56 residue has been established as the central target for the identification of 1A-116 as Rac1 inhibitor (Gao et al., 2001).

To further evaluate the critical role of W56 in the Rac1-1A-116 interaction, several docking experiments were performed using Rac1 (1MH1A) and CDC42 (2QRZB) PDB structures as receptors. Additionally, we also carried out *in silico* experiments using single-point mutants, replacing W56 in Rac1 for phenylalanine residue and replacing the F56 residue of Cdc42 for a tryptophan. Rac1 W56F and CDC42 F56W 3D models were generated with the I-TASSER software (Figures 3B,C). As seen in Figure 3D, when 1A-116 is docked to Rac1 W56F, the compound affinity for the GTPase decreases, showing a docking energy of −6.08 ± 0.226 kcal/mol (vs. −5.59 ± 0.0139 kcal/mol of the wild-type Rac1). Furthermore, when the affinity of 1A-116 for Cdc42 and Cdc42 F56W was evaluated, a clear increase of its binding affinity is observed (from -5.69 ± 0.0170 to -6.09 ± 0.00994 kcal/mol, respectively. The absence of W56 in Rac1 structure or mutant Cdc42 causes the loss of the H-bond between 1A-116 and the protein as shown in Figures 3B,C. In summary, we validated the relevance of the H-bond established between 1A-116 and W56 of Rac1 for this drug-protein interaction *in silico*.

We then evaluated the specificity of 1A-116 for the W56 site *in vitro*. For this purpose, we carried out site-directed mutagenesis to generate the two mutant versions of Rac1 and Cdc42 to recapitulate the *in silico* experiments. We used the SRE-Luc reporter system, containing the serum response element (SRE) fused to the luciferase gene to evaluate the effects of these mutations in 1A-116 biological activity. At day zero, COS-1 cells were co-transfected with Rac1 and Cdc42 plasmids (and carried out an SRE-Luc activation assay in the presence or absence of 1A-116 for 24 h, together with the reporter plasmids SRE-Luc and Renilla. As negative control, we used cells transfected with the empty expression vector (EV). As shown in Figure 3E, 1A-116 inhibits SRE-activation mediated by Rac1 by 40%. However, this inhibition is lost in the case of the W56F mutant version of Rac1. We also observed that 1A-116 could not inhibit the Cdc42-mediated activation of SRE. However, it does so in the case of cells expressing the Cdc42 F56W mutant. (Figure 3E). These findings support the idea that the W56 residue of Rac1 is necessary for the inhibitory activity of 1A-116.

Finally, we studied the effect of 1A-116 in a rapid nucleotide cycling mutant of Rac1 that bears the P29S mutation (Figure 3A, shaded area). We first evaluated the interaction of 1A-116 with this mutant using an *in silico* docking strategy with the crystal structure of this mutant protein (3SBDA PDB structure). We found that the predicted affinity of 1A-116 for this mutant is similar to that calculated with the Rac1 wild-type structure (1MH1A) (-6.18 ± 0.0402 kcal/mol) (Figure 3D). To study the effect of 1A-116 on this mutant *in vitro*, we generated a double Rac1 mutant harboring both the P29S and the Q61L substitutions and test its activity on SRE experiments in the presence and absence of the inhibitor. As shown in Figure 3E, 1A-116 is also able to inhibit SRE-activation mediated by both P29S mutants, indicating that 1A-116 can inhibit the gain-of-function mutation of Rac1. Figure 3F shows western blot expression detection of transfected exogenous proteins.

### 1A-116 Inhibits the SRE-Activation Mediated by Different GEFs

**FIGURE 4:**
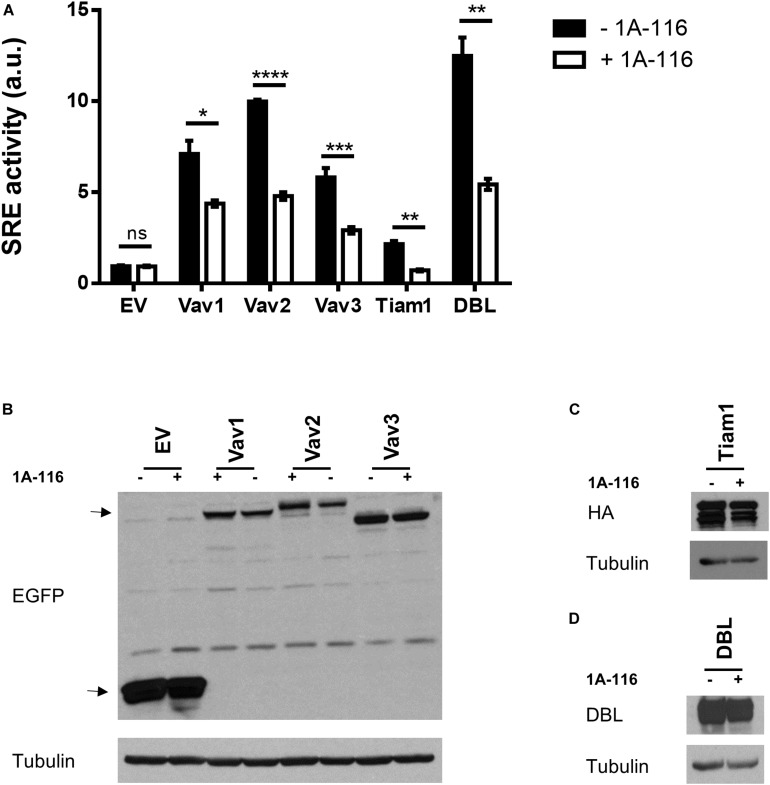
1A-116 inhibits SRE-activation mediated by different GEFs. **(A)** COS-1 cells were co-transfected with active versions of different GEFs together with SRE-Luc and Renilla reporter plasmids, treated (+1A-116, white) or not (-1A-116, black) with 1A-116 50 μM and luciferase activity was measured. **(B–D)** Western blot expression detection of transfected exogenous proteins. The expressed protein is reported and the primary antibodies used (WB) were: α-EGFP for EV, Vav1 Δ1-189, Vav2 Δ1-186 and Vav3 DH-PH-ZF; α-HA for Tiam1 C1199; α-DBL for oncogenic DBL and α-Tubulin as a loading control. Error bars, SEM *t* test; ns, not statistically significant; *p < 0.05; **p < 0.01; ***p < 0.001; ****p < 0.0001; vehicle vs. 1A-116 for each protein. Luciferase expression was relativized to Renilla expression value (transfection control) and values reported are shown as an increase in activity with respect to the mean luciferase expression of the empty vector (EV), in arbitrary units (a.u.). Results are representative of at least three independent experiments.

To identify which interactions between Rac1 and different GEFs were inhibited by 1A-116, we evaluated the effect of the 1A-116 inhibitor in the SRE activity elicited by a number of activated versions of Rac1 GEFs when ectopically expressed in COS1 cells. Those activated GEFs included Vav1 Δ1-189, Vav2 Δ1-186, Vav3 DH-PH-ZF, Tiam1 C1199 (aa 391-1590) and oncogenic Dbl (through truncation of the N-terminal 497 residues). We found that 1A-116 also inhibits the activation of all those GEFs, with percentages of inhibition of 40-50% (Vav family GEFs), 60% (Dbl) and 75% (Tiam1) (Figure 4A). Figures 4B–D show western blot expression detection of transfected exogenous proteins.

### 1A-116 Inhibits Rac1 Activity at the GEF-Rac1 Level

In order to shed light on the hypothesis that 1A-116 inhibits Rac1 activity at the GEFs-Rac1 interaction level, we carried out a 3D differentiation assay using a human keratinocytes cell line and different pull-down assays, using two different experimental schemes.

**FIGURE 5:**
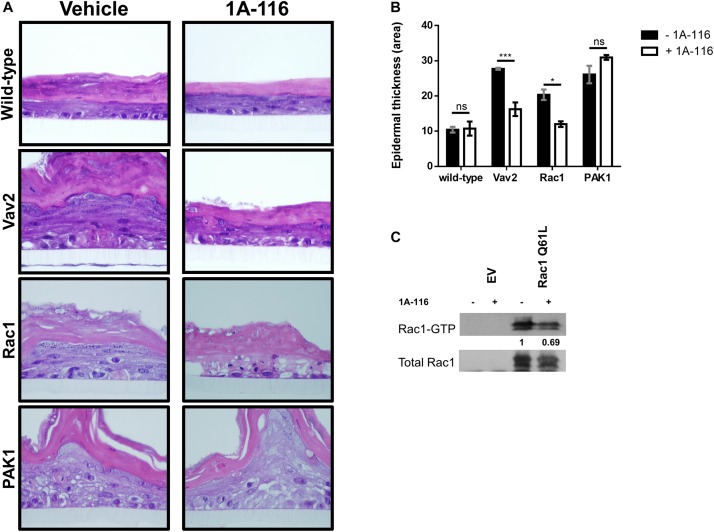
1A-116 acts by inhibiting Rac1 activity at the GEFs-Rac1 level. **(A)** Representative histological sections of 3D organotypic cultures of human keratinocytes expressing the indicated proteins (left), treated or not with 1A-116 100 nM. **(B)** Quantification of the epidermal thickness area using data from panel **A**. *t* test; ns, not statistically significant; *p < 0.05; ***p < 0.001. **(C)** Transfected COS-1 cells Rac1 Q61L were treated with vehicle (-) or with 1A-116 50 μM (+). Cell lysates were precipitated by pull-down assay with GST-PAK1. Densitometric values are reported (arbitrary units taking Rac1 Q61L treated with vehicle as 1). The western blot analysis was carried out using an anti-Rac1 antibody.

First, we used stably transfected Ker-CT human keratinocytes cell lines with active versions of different components of the Rac1 signaling pathway: a GEF-type activator (oncogenic Vav2), a fast cycling version of Rac1 (Rac1 F28L) and a direct constitutively active effector of Rac1 (PAK1 Tyrosine 423). The assay was carried out by culturing the cells on polycarbonate inserts, treated or not with 1A-116 at concentrations in the nanomolar range (100 nM). Ker-CT wild type cells growing under these conditions developed a distinctive stratified epidermal architecture composed of proliferative basal and suprabasal differentiated keratinocytes, as well as a superficial stratum corneum (Figure 5A; wild-type, vehicle panel). This 3D organotypic structure did not suffer anyalteration when treated with 1A-116 (Figure 5A; wild-type, 1A-116 panel). The stable overexpression of different active components of the Rac1 signaling pathway caused the development of hyperplasia and the formation of a disorganized and invasive epithelium (Figure 5A; Vav2, Rac1, PAK1, vehicle panel). These cells also form thicker epidermal layers than wild-type cells, under the same conditions (Figure 5B). However, when treated with 1A-116, this phenotype was reversed in the case of Vav2 and Rac1-mediated hyperplasia, decreasing the thickness of the epidermal layers (Figure 5B), but not in the PAK1-mediated one (Figure 5A, Vav2, Rac1 and PAK1, 1A-116 panel). In the case of pull-down assay, COS-1 cells were transfected with Rac1 Q61L that were either treated or untreated with 1A-116 for 24 h. We carried out the pull-down, showing a decrease in Rac1-GTP levels by 1A-116 (Figure 5C). This result indicates that the Rac1-GTP levels have not been altered by the inhibitor, because the activation by GEFs was not taking place since we only studied the interaction of 1A-116 with the already active protein lysate.

Taking the 3D differentiation assay and *in vitro* pull-down assay together, these results corroborate the hypothesis that 1A-116 is acting at GEFs-Rac1 interaction level.

### 1A-116 Inhibits Rac1-Regulated Processes, Like Ruffles and Lamellipodia Formation

**FIGURE 6:**
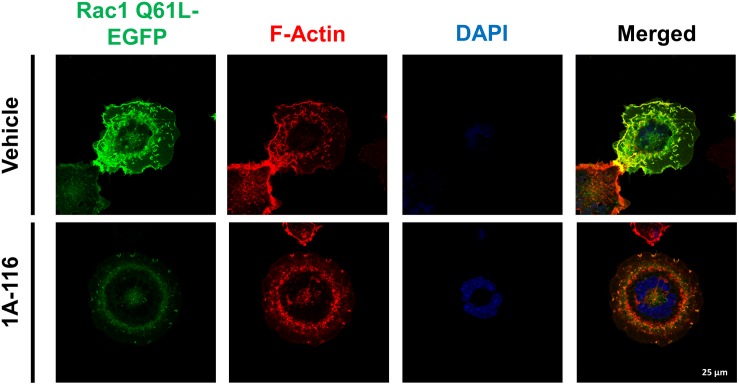
1A-116 inhibits formation of ruffles and lamellipodia, cellular processes regulated by Rac1. COS-1 cells transfected with Rac1 Q61L fused to EGFP (Rac1 Q61L-EGFP) were cultured in poly-lysine coated glasses and treated with 1A-116 50 μM or not (vehicle) for 24 h. Cells were fixed and stained with AlexaFluor555-phalloidin and DAPI (to observe the actin filaments and nuclei, respectively) and were observed by confocal microscopy (63×).

Finally, we evaluated the effect of 1A-116 on the rearrangement of cytoskeleton induced by Rac1, by transfecting COS-1 cells with Rac1 Q61L version fused at its N-terminal with EGFP. After 24 h, we subcultured and seed the cells in poly-lysine coated glass and treated them for 16 h with 1A-116. Then, we fixed and stained cells with AlexaFluor555-phalloidin and DAPI and analyzed them by confocal microscopy. As shown in Figure 6 (vehicle, upper row), Rac1 Q61L expression generated peripheral ruffles formation, which co-localize with Rac1. However, this phenotype was abolished when cells were treated with 1A-116 (Figure 6, 1A-116, bottom row), showing once again that this compound inhibits cellular processes involved in the metastatic cascade and regulated by Rac1.

## Discussion

The elucidation of the specific mechanism of action associated with novel therapeutic compounds is a crucial component of the drug discovery process. Given the high attrition rates in drug discovery, it is of great interest having a deep understanding of drug activities before exploring the clinical benefit of these molecules. Here, we provide data regarding the pharmacology of the Rac1 inhibitor, 1A-116, a small molecule developed by our group that has already shown promising preclinical performance (Cardama et al., 2014a, b; Cabrera et al., 2017; Gonzalez et al., 2017). For this purpose, we conducted an interdisciplinary approach taking advantage of bioinformatics, confirming this information with *in vitro* testing and finally, analyzing different events in cell-based assays where Rac1 plays a vital role.

1A-116 compound is a PPI inhibitor developed by our group using a rational design approach. Targeting PPIs has been a neglected strategy for many years; however, it has become clear that some regions are more critical for protein binding than others, in the large interfaces spanning these PPIs. This provides the opportunity to determine which few residues contribute significantly to the free energy of binding between interacting proteins and to design small molecules able to block those residues involved. The 1A-116 compound was first identified using a docking-based virtual screening approach (Cardama et al., 2014a), based on the knowledge that Rac1 possesses a particular area in its structure responsible for interacting with GEF-type activators (Gao et al., 2001). Of interest, one particular residue, the tryptophan (W56), seems to be a significant determinant of this PPI and this particular residue was placed as the target to conduct the virtual screening. At that time, a single crystallographic structure was used in the structure-guided design of this GEF-Rac1 inhibitor. However, this method did not take into account the possibility of substantial flexibility of the protein structure and only addressed the selected binding site within the protein as a static or rigid structure. It has been shown that protein flexibility is a key component to take into consideration for drug design (Teague, 2003; Arkin and Wells, 2004). To evaluate how protein flexibility affects Rac1-1A-116 interaction, we took advantage of all the Rac1 crystal structures available and deposited in the PDB to span the spectrum of possible conformations. The structural differences between these conformers characterize the conformational diversity of the protein (Palopoli et al., 2016) and we hypothesized that these differences might have a profound impact on protein function and 1A-116 activity.

Experimentally, the differences between structures can be determined using the RMSD score distribution of all conformers. The analysis of Rac1 conformational diversity shows that while globally Rac1 has a flexible structure (RMSD of maximum pair = 2.5 Å), the residues engaged by 1A-116 binding, and specifically the W56, have a *Z*-Score below zero, showing relatively low mobility within protein structure. This stable region of the protein allows 1A-116 to effectively bind to Rac1; taking into account the flexibility of the protein. Rac1, like other GTPases, can undergo conformational changes depending on their union with GTP or GDP. However, our results analyzing the *Z*-score per residue taking into account all the Rac1 conformers available in the PDB show that the amino acids involved in the interaction with the compound correspond to a zone of low mobility beyond the nucleotide to which it is bound. This concept does not contradict the proposed mechanism of action for the compound, meaning the interference of the Rac1-GEF interaction. This highlights W56 and the surrounding residues as an interesting and stable zone for targeted therapies taking into account the conformational diversity of Rac1. Furthermore, through the re-docking analysis of 1A-116 to the maximum RMSD pair of conformers, we were also able to determine the nature of the interaction of 1A-116 with Rac1. In this sense, 1A-116-Rac1 interaction is predominantly achieved through the 3,5-dimethylphenyl group (Ring A) and the H-bond established by the guanidine with the W56. Moreover, the 2-trifluoromethylphenyl group rotatable bonds allows 1A-116 to adopt the most favorable pose to each conformer with no loss of binding affinity.

In addition, W56 residue resides in a highly conserved region of the GTPases that belong to the Ras superfamily; e.g., W56 in Rac1 corresponds to L56 in Ras GTPase. In fact, L56 in Ras oncoprotein has been described as one of residues surrounding the binding pocket of Ras small molecule inhibitors (Maurer et al., 2012; Schöpel et al., 2016) or even being one of the residues being bound by the inhibitor (Cruz-Migoni et al., 2019).

It is important to emphasize that solely the ability for a ligand to bind to a certain binding site, does not make a compound a suitable inhibitor with therapeutic perspectives: “drug-likeness” is one key determinant for drug discovery process. In this regard, 1A-116 meets all the criteria of drug-likeness stated by Lipinsky and Veber (Lipinski et al., 2001; Veber et al., 2002; Lipinski, 2004) as shown here, making it possible to formulate an oral vehicle for it.

Even though the initial virtual screening was designed to identify W56-interacting small molecules, we show here the critical role of W56 in 1A-116 binding. Since Cdc42 Rho GTPase presents a 70% sequence identity with Rac1 and has differences in key residues such as a phenylalanine in the 56 position, docking experiments were carried out. These experiments show increased binding energy for 1A-116 docked to Cdc42 compared to its binding energy to Rac1. This reduction in binding affinity seems to be due to the loss of the H-bond established between 1A-116 and the W56 residue of Rac1. Moreover, we also docked 1A-116 to a Rac1 W56F mutant and to a Cdc42 F56W mutant showing a striking congruence, spotlighting W56 as the key residue for 1A-116 binding. The 3D-structure of Rac1 in complex with Tiam1 GEF showed that W56 generates hydrogen-bond type interactions with the histidine 1178 of Tiam1, also establishing numerous van der Waals interactions with nearby amino acids (Gao et al., 2001). Substitution of W56 by phenylalanine causes a lower occupation of the hydrophobic pocket of Rac1 (due to the smaller size of phenylalanine), with the consequent loss of interactions with the amino acids of the GEF. Collectively, these results prompted us to explore the biochemical significance of W56 residue *in vitro*.

Using a widely used bioluminescent luciferase reporter system based on the serum response element (SRE), we explored the effect of 1A-116 on Rac1 and Cdc42 GTPases activities. Of note, SRE reporter system is regulated by different members of Rho GTPase family of proteins and presents interesting features such as sensitivity, wide dynamic range and lack of endogenous activity that makes it a good reporter system to evaluate the *in vitro* activity of 1A-116 (Montaner et al., 1999). 1A-116 effectively inhibited Rac1 activity and did not affect Cdc42-mediated SRE activation *in vitro*, results that were previously shown using pull down assays (Cardama et al., 2014a). These SRE-Luc experiments were carried out using the Q61L mutant of Rac1 in order to increase the sensitivity of the reporter system. Eventhough this mutant version is associated to a constitutive activity, it retains GEF and/or GAP mediated regulation. This has already been shown in mutant versions of other GTPases, such as Ras oncoprotein, where the mutation causes the loss of interaction with GAP proteins. Additionally, it has been recently demonstrated that novel compounds designed block the G12C K-Ras mutant surprisingly exhibit certain dependency to nucleotide exchange promoted by GEF activity (Ostrem et al., 2013).

Additionally, Rac1 W56F and Cdc42 F56W mutants developed by site-directed mutagenesis showed a close dependency of W56 presence in Rac1-GEF binding pocket. Taking into account these results, we postulate that 1A-116 prevents the correct positioning of the GEF, inhibiting the activation of Rac1. These experimental data shows a correlation between *in silico* and *in vitro* testing, pointing out to the crucial role of W56 on 1A-116 binding and activity.

Recently, two independent whole-exome sequencing studies revealed a novel gain-of-function mutation of Rac1 in sun-exposed melanomas, being the most frequently observed somatic mutation after BRAF and NRAS mutations (Hodis et al., 2012; Krauthammer et al., 2012). Of interest, Rac1 P29S mutant has been identified as an essential growth driver that promotes cell proliferation, confers resistance to BRAF inhibitors and may be involved in immune escape by enhancing PD-L1 expression (Watson et al., 2014; Vu et al., 2015). We evaluated 1A-116 binding to Rac1 P29S mutant *in silico* and using the SRE-luciferase reporter system. In both cases, 1A-116 was able to inhibit Rac1 P29S activation, showing the potentiality to use 1A-116 compound in Rac1 P29S-driven tumors (Davis et al., 2013). Further studies are required to determine the therapeutic efficacy, but it may represent an interesting precision medicine strategy for melanoma treatment.

Although the presence of the Rac1 mutation in melanoma, it is more often that tumors show altered expression and/or mutations in upstream regulatory proteins, such as GEFs (Vigil et al., 2010). It has been shown that different groups of GEFs are relevant in different tumor types; therefore, we evaluated the effect of 1A-116 on SRE activation by a group of different constitutively active GEFs. We tested the activity of the members of the Vav family (Vav1, Vav2, and Vav3), Tiam1 and DBL and 1A-116 was able to block the activity of all these activators in the SRE-luc reporter assay. Vav family of GEFs are required for the development of breast cancer, leukemia and skin cancer (Chang et al., 2012; Citterio et al., 2012; Menacho-Márquez et al., 2013), while Tiam1 overexpression correlates with tumor progression in pancreatic cancer, breast cancer and colorectal cancer (Li et al., 2016; Ding et al., 2018; Izumi et al., 2019).

Rac1 has previously been shown to be involved in the defective activation of several signaling cascades leading to anomalous behavior of cells and ultimately contributing to cancer progression. Moreover, Rac1 is involved in epithelial-mesenchymal transition (EMT), a key process in the metastatic cascade (Lv et al., 2013; Nakaya et al., 2004). Metastasis is the end of a very complex multistep process where cancer cells migrate from their primary site and colonize other organs. It accounts for 90% of cancer deaths (Lyden et al., 2011). Rho GTPases have an essential role during the metastatic cascade. Rac1, in particular, contributes to cancer development, stimulating cell proliferation and loss of cell polarity (Ellenbroek and Collard, 2007) and by altering cell-to-cell and cell-to-matrix junctions, it promotes migration and invasion to distant sites.

Rac1 also regulates cytoskeleton reorganization, and promotes the formation of cell surface extensions like lamellipodia, a classical feature of mesenchymal movements (Parri and Chiarugi, 2010). The invasive phenotype of metastatic cancer cells causes the remodelation of extracellular matrix by producing metalloproteases, key components of the EMT also regulated by Rac1 (Ellenbroek and Collard, 2007; Bosco et al., 2009).

The central role of the Rac1 pathway in the metastatic phenotype was well demonstrated by different studies. It has been shown a direct relationship between Rac1 activation and the metastatic potential of breast cancer cells (Baugher et al., 2005). Moreover, Rac1 activity and increased levels of PAK1 expression were associated with lymph nodes metastasis in urothelial carcinoma (Kamai et al., 2010). Finally, GEFs like VAV2 showed to be important in squamous carcinomas of the head and neck (Patel et al., 2007) and VAV3 in glioblastoma and breast cancer (Chan et al., 2005). Depletion or inhibition of these GEFs impaired cell migration, invasion and proliferation.

Using different cancer models, we have already validated Rac1 as a target and 1A-116 as a small molecule to be potentially exploited in therapeutics schemes (Cardama et al., 2014a; Cabrera et al., 2017; Gonzalez et al., 2017). Of interest, Rac1 has been shown to have a vital role in skin physiology (Benitah et al., 2005; Castilho et al., 2010) and Rac1 hyperactivation drives pathologic conditions, promoting proliferation of keratinocytes and immune infiltrate (Chen et al., 2014). Indeed, Rac1 showed a hyperproliferative-specific function in a genetically engineered keratinocyte restricted Rac1 deletion mouse model (Wang et al., 2010), and Rac1 expression was found to be elevated in papillomas and squamous cell carcinomas (Benitah et al., 2005). We showed here that 1A-116 could inhibit keratinocytes hyperplasia, reducing invasive phenotype and tissue disorganization. This hyperplastic 3D model was established by stably transfecting human Ker-CT cells with different members of Rac1 pathway. In all the cases, the organotypic 3D keratinocyte culture showed a hyperproliferative phenotype compared to the control cells but only GEF-driven (Vav2) and Rac1-driven hyperplasia were reversed by 1A-116 treatment.

Additionally, we performed pull down assays showing that 1A-116 interferes with Rac1 activation. Collectively, these results are consistent with the initial rational design of 1A-116 as a GEF-Rac1 PPI inhibitor and show that 1A-116 is able to reduce Rac1 signaling pathway at the nanomolar range of concentration in a 3D skin model. Nevertheless, these results do not discard the possibility that 1A-116 interaction with W56 residue could affect Rac1 affinity to some effectors such as PLC-γ2 (Jezyk et al., 2006; Bunney et al., 2009) and contribute to 1A-116 effect. However, 1A-116 has no effect whatsoever on PAK1 activity, as shown throughout our work. Rac1 has been historically linked to actin dynamics regulation, and 1A-116 can interfere with this activity. In this regard, we also showed that 1A-116 negatively affects the formation of different actin-based structures such as membrane ruffles and lamellipodia. These results show that 1A-116 is able to modulate Rac1-mediated processes such as actin dynamics and keratinocyte hyperproliferation.

As we mentioned above, the Rac1 pathway, including both upstream activators and downstream effectors, is a critical player in the invasive and metastatic phenotype. This has been shown in a great variety of tumor types and gives the basis to consider Rac1 as an attractive and validated target to develop molecular therapies against cancer metastasis. Therefore, our results support a critical role of W56 residue of Rac1 in 1A-116 activity. We validated by *in silico* and *in vitro* approaches that 1A-116 is only able to exert its activity when W56 is present in the protein structure. Further, 1A-116 showed its ability to interfere in different Rac1-mediated biological processes under different experimental settings, including a hyperproliferative 3D keratinocyte model. Importantly, 1A-116 is able to interfere with Rac1 P29S mutant activity, and this may provide an interesting therapeutic strategy for melanoma patients with a particular mutation profile. Taken together, we can conclude that 1A-116 is a PPI inhibitor able to selectively bind to W56 residue in Rac1 protein structure and may represent a suitable therapeutic agent for different types of neoplasms as well as pre-malignant disorders involving hyperproliferative phenotypes.

## Data Availability Statement

All datasets generated for this study are included in the article/Supplementary Material.

## Author Contributions

NG participated in all experimental work, analyzed data, and contributed to both artwork design and manuscript writing. GC contributed to both analyzed data and manuscript writing. PL and PC performed the docking experiments. JR-V and SR-F contributed to the design and experimental work of luciferase and site-directed mutagenesis assays. LL-M contributed to the design and experimental work of the 3D skin model assay. XB conceived the luciferase and 3D skin model assays and analyzed data. PL and DG conceived the work, analyzed data, contributed to manuscript writing and performed the final editing of the text.

## Conflict of Interest

GC and PL served in a consultant/advisory role for Chemo- Romikin S.A. The remaining authors declare that the research was conducted in the absence of any commercial or financial relationships that could be construed as a potential conflict of interest.
