# Supplementary material for: Computational and in vitro Pharmacodynamics Characterization of 1A-116 Rac1 Inhibitor: Relevance of Trp56 in Its Biological Activity
Source: Front Cell Dev Biol. 2020 Apr 15;8:240. doi: 10.3389/fcell.2020.00240 (PMC7174510; doi:10.3389/fcell.2020.00240)
Supplement: Supplementary file 1 [file Table_1.pdf]

Table 1: Rac1 fifty-two (52) conformations used for computational conformational analysis and Docking experiments

Conformer	Resolution	Affinity for 1A-116	Co-cristalized with	Crystalized Ligand	Aditional data
1G4UR	2.3 Å	-6.1 kcal/mol	TYROSINE PHOSPHATASE OF SALMONELLA	GDP	-
1HE1C	2 Å	-5.9 kcal/mol	EXOENZYME PSEUDOMONA	GDP	-
1HE1D	2 Å	-6.1 kcal/mol	EXOENZYME PSEUDOMONA	GDP	-
1HH4A	2.7 Å	-5.8 kcal/mol	RHO	GDP	-
1HH4B	2.7 Å	-6.3 kcal/mol	RHO	GDP	-
1I4DD	2.5 Å	-6.4 kcal/mol	ARFAPTIN 2	GDP	-
1I4LD	2.7 Å	-6.5 kcal/mol	ARFAPTIN 2	GDP	-
1RYFA	1.75 Å	-5.0 kcal/mol	-	GDP	Self-activating isoform
1RYFB	1.75 Å	-5.4 kcal/mol	-	GDP	Self-activating isoform
2H7VA	2.6 Å	-6.5 kcal/mol	PROTEIN KINASE ypkA	GDP	-
2H7VB	2.6 Å	-5.8 kcal/mol	PROTEIN KINASE ypkA	GDP	-
2P2LA	1.9 Å	-6.3 kcal/mol	-	GDP	Trimer displaces Mg for Zn
2P2LB	1.9 Å	-6.2 kcal/mol	-	GDP	Trimer displaces Mg for Zn
2P2LC	1.9 Å	-6.5 kcal/mol	-	GDP	Trimer displaces Mg for Zn
5N6OA	2.59 Å	-6.1 kcal/mol	-	GDP	-
5N6OB	2.59 Å	-5.9 kcal/mol	-	GDP	-
5O33A	1.64 Å	-5.6 kcal/mol	KALIRIN	GDP	-
1E96A	2.4 Å	-6.1 kcal/mol	NCF2	GTP	-
1I4TD	2.6 Å	-6.4 kcal/mol	ARFAPTIN 2	GNP	Non-hydrolysable GTP analogue

1MH1A	1.38 Å	-6.0 kcal/mol	-	GNP	Non-hydrolysable GTP analogue
1RYHA	1.75 Å	-5.3 kcal/mol	-	GNP	Self-activating isoform
1RYHB	1.75 Å	-5.3 kcal/mol	-	GNP	Self-activating isoform
2FJUA	2.2 Å	-6.0 kcal/mol	PLCB2	GSP	GTP analogue
3SBDA	2.1 Å	-6.2 kcal/mol	-	GNP	Mutant - P29S, spontaneously activated; non-hydrolysable GTP analogue
3SBDB	2.1 Å	-6.0 kcal/mol	-	GNP	Mutant - P29S, spontaneously activated; non-hydrolysable GTP analogue
3SBEA	2.6 Å	-6.2 kcal/mol	-	GNP	Mutant - P29S, spontaneously activated; non-hydrolysable GTP analogue
3SUAA	4.39 Å	-6.2 kcal/mol	PLEXIN B1	GNP	Non-hydrolysable GTP analogue
3SUAB	4.39 Å	-6.1 kcal/mol	PLEXIN B1	GNP	Non-hydrolysable GTP analogue
3SUAC	4.39 Å	-6.1 kcal/mol	PLEXIN B1	GNP	Non-hydrolysable GTP analogue
3TH5A	2.3 Å	-6.2 kcal/mol	-	GNP	Non-hydrolysable GTP analogue
3TH5B	2.3 Å	-6.0 kcal/mol	-	GNP	Non-hydrolysable GTP analogue
4GZLA	2 Å	-6.0 kcal/mol	-	GNP	Mutant - Q61L, constitutively active
4GZLB	2 Å	-6.1 kcal/mol	-	GNP	Mutant - Q61L, constitutively active
4GZMA	2.8 Å	-5.8 kcal/mol	-	GSP	Mutant - F28L, spontaneously activated
4GZMB	2.8 Å	-5.8 kcal/mol	-	GSP	Mutant - F28L, spontaneously activated
6BC1A	2.9 Å	-5.9 kcal/mol	RHO	GSP	GTP analogue
6BC1B	2.9 Å	-5.9 kcal/mol	RHO	GSP	GTP analogue

1FOEB	2.8 Å	-5.8 kcal/mol	TIAM1	-	No ligand cristalized
1FOED	2.8 Å	-5.8 kcal/mol	TIAM1	-	No ligand cristalized
1FOEF	2.8 Å	-5.8 kcal/mol	TIAM1	-	No ligand cristalized
1FOEH	2.8 Å	-5.7 kcal/mol	TIAM1	-	No ligand cristalized
2NZ8A	2 Å	-5.7 kcal/mol	TRIO	-	No ligand cristalized
2VRWA	1.85 Å	-5.9 kcal/mol	VAV	-	No ligand cristalized
2YINC	2.7 Å	-6.4 kcal/mol	DOCK2	-	No ligand cristalized
2YIND	2.7 Å	-6.0 kcal/mol	DOCK2	-	No ligand cristalized
3B13B	3.006 Å	-6.4 kcal/mol	DOCK2	-	Mutant - T17N, dominant negative mutant; no ligand crystalized
3B13D	3.006 Å	-6.3 kcal/mol	DOCK2	-	Mutant - T17N, dominant negative mutant; no ligand crystalized
4YONB	1.95 Å	-6.2 kcal/mol	PREX	-	No ligand cristalized
5FI0B	3.282 Å	-6.1 kcal/mol	PREX	-	No ligand cristalized
5FI0D	3.282 Å	-6.3 kcal/mol	PREX	-	No ligand cristalized
5FI0F	3.282 Å	-6.3 kcal/mol	PREX	-	No ligand cristalized
5FI0H	3.282 Å	-6.1 kcal/mol	PREX	-	No ligand cristalized

Table 1. This table shows information about the fifty-two (52) human Rac1 conformations analyzed in this study. All X-ray structures were retrieved from the Protein Data Bank (13). In this table: GTP stands for Guanosine-5'-triphosphate; GDP for Guanosine-5'-diphosphate; GNP for Phosphoaminophosphonic acid-guanylate ester; and GSP for 5'-Guanosine-diphosphate-monothiophosphate.
